# Prevalence and associated factors of unintended pregnancy among pregnant woman in Gondar town, North west Ethiopia, 2014

**DOI:** 10.1186/s13104-019-4203-6

**Published:** 2019-03-22

**Authors:** Fentahun Yenealem, Gedefaye Niberet

**Affiliations:** 1 Department of Midwifery, College of Medicine and Health Science, Bahir Dar University, Bahir Dar, Ethiopia; 2 Department of Midwifery, College of Medicine and Health Science, Debre Tabor University, Gondar, Ethiopia

**Keywords:** Unintended, Pregnancy, Gondar town

## Abstract

**Objective:**

The purpose of this study was to assess prevalence and associated factors of unintended pregnancy among pregnant women in Gondar town, North western Ethiopia, 2014. A community based cross-sectional study was conducted among pregnant women to select 325 participants for face to face interview by using simple random sampling technique from April 1–May 30, 2014. Bivariate and multivariate data analysis was performed using SPSS for Windows version 20 and level of significance of association was determined at *P* value < 0.05.

**Result:**

This study identified that 20.6% of pregnant women were unintended of which 6.8 were mistimed and 13.8 were unwanted. Unintended pregnancy was associated with family size (≥ 4) (AOR = 2.92; 95% CI 1.605, 5.31), marital status (single) (AOR = 12.59; 95% CI 5.18, 30.6) and age at first pregnancy < 18 years AOR (95% CI) 3.02 (1.522, 6.245). Therefore it is important to adequately counsel women concerning positive mind-sets about its prevention mechanism and its consequences of unintended pregnancies.

## Introduction

Unintended pregnancy was identified as either mistimed or unwanted at a time of conception [1, 2]. Unintended pregnancy is a pregnancy that was not wanted at the time conception occurred, irrespective of whether or not contraception was being used [3].

Unintended pregnancy is a worldwide problem that affects women, their families and societies at large. Unintended pregnancy can result from not using contraceptives, contraceptive failure and also, less commonly, from rape [4].

It is a public health problem which affects maternal and child health like, abortion, low birth weight baby; preterm birth and high infant and maternal mortality are attributed to unintended pregnancies [5, 6]. Of the estimated 210 million pregnancies that occur throughout the world each year, about 38% are unplanned, out of which 22% end in abortion [7]. Ninety-five percent of unsafe abortions occur in developing countries. Millions more suffer long-term injuries from often life-threatening complications. In many poor countries, treatment of these complications consumes up to half of hospital budgets for obstetric. In many poor countries, treatment of these complications consumes up to half of hospital budgets for obstetrics and gynecology [8].

It was estimated that current levels of unintended pregnancy, the prevalence of contraceptive use and the number of unintended pregnancies stem from early discontinuation and typical method failure rates. Every year in sub-Saharan Africa, approximately 14 million unintended pregnancies occur and a sizeable proportion is due to poor use of short-term hormonal methods [9]. According to the World Health Organization (WHO), the country has the fifth largest number of maternal deaths in the world [10]. In Ethiopia, according to the 2011 EDHS, 25% of women with births in the 5 years before the survey and 32% of current pregnancies were reported as unintended [11]. Therefore we intended to assess the prevalence and its associated factors of unintended pregnancy.

## Main text

### Methods

#### Study setting and period

Community based cross sectional study was conducted from April 1 to May 30, 2014 among pregnant women in Gondar town. Gondar town which is located the North Gondar administrative, North western Ethiopia. The town is located at an altitude 12.4N and 27.21E. It is 182 km away from regional town Bahir Dar and 748 km from capital city Addis Ababa. The town has 13 urban 7 rural administrative kebeles. In the town around 248,784 total projected populations were presented in the year 2010/2011; among these around 131,111 were females [11].

#### Sample size and sampling techniques

The sample size required for assessment of the prevalence of unintended pregnancy and associated factors among pregnant women in Gondar town was calculated using single population proportion formula by considering the following assumption: proportion (**p**) = 27.9% [12], **w** = tolerable (margin) of error = 5% and **Z** = **Z** score for 95% confidence interval = 1.96 then the final calculated sample size was 325.

Sampling frame was collected from health extension workers who work in 20 urban and rural kebeles. From 20 kb there were around 643 pregnant women documented in the health extension report in the half year 2014; from these we generate the frame and 325 pregnant women recruited for face to face interview by using simple random sampling technique.

#### Measurement

Data were collected through face to face interviews using a structured and pre-tested questionnaire. The questionnaire was first prepared in English then translated to Amharic and back to English again by language expert in order to maintain the consistency of the instrument. A pre-test was conducted on 16 pregnant mothers in one of the kebeles out of the study area before the main and the instrument was amended accordingly. Five diploma nurses had conducted the face to face interviews and two B.Sc. degree Midwives had supervised the data collection process. Training was given to the data collectors and supervisors before the actual data collection regarding the aim of study, data collection tool and procedures.

The information was collected on socio demographic data (mothers’ age, mother’s age at marriage, marital status, place of residence, family income, educational status, occupation, educational status, family size); reproductive variables such as: previous ANC visit, parity, gravidity, history of abortion and still birth, state of pregnancy (intended or unintended), contraceptive utilization before current pregnancy, age at first pregnancy were included.

#### Statistical analysis

The data was cleaned, coded, entered into EPI Info version 3.5.3 and exported to SPSS version 20 software package for analysis. The data first was analyzed using bivariate logistic regression and then all explanatory variables which had P-value ≤ 0.05 were entered into multivariate logistic regression model to determine the effect of various factors on the dependant variable and to resolve confounding effects. Association between dependent and predictor variables was assessed using AOR and 95% CI. Statistical significance was declared when the P-value was less than 0.05.

#### Operational definition

Unintended pregnancy either mistimed or unwanted at a time of conception [1].

### Results

#### Socio-demographic and reproductive characteristics of the respondents

A total of 325 women were interviewed with response rate of 100% of these 73.8%) were age in the group of 20–29 years with a mean age of 26.16 (SD ± 4.79) years. Two hundred fifty-eight (79.4%) of the study subjects had a family size of 1–4. Two-hundred sixty-nine (82.8%) were orthodox in religion and 312 (96%) were Amhara in ethnic group. Seventy-four (22.8%) were not attend formal education and 96 (29.5%) attended primary school (Table 1).

**Table 1:** Socio-demographic and reproductive characteristics of pregnant women in Gondar town, Northwestern Ethiopia, 2014

Variable	Frequency	Percent
*Age, years*		
15–19	18	5.5
20–24	119	36.6
25–29	121	37.2
30–34	57	17.3
35–49	10	3.1
*Family size*		
< 4	199	61.2
> 4	126	38.8
*Religion*		
Orthodox	269	82.8
Muslim	47	14.5
Others	9	2.7
*Education*		
Unable to read and write	51	15.7
Able to read and write	23	7.1
Elementary (1–8)	96	29.5
High school (9–12)	117	36.00
Higher education	35	11.7
*Occupation*		
House wife	203	62.5
Governmental employee	37	11.4
Self employee	49	15.1
Daily laborer	13	4
Others^a^	23	7.1
*Ethnicity*		
Amhara	312	96
Others	13	4
*Age at first pregnancy, years*		
< 18	120	36.9
≥ 18	205	63.1
*Number of pregnancy*		
1–2	223	74.8
3–4	69	21.2
≥ 5	13	4
*Previous ANC (n =* *137)*		
Yes	130	95
No	37	5
*Pre pregnancy contraceptive use*		
Yes	253	77.8
No	72	22.2
*Marital status*		
Single	27	8.3
Married	298	91.7

^a^Student, voluntary work, religious work

#### Current state of pregnancy

**Fig. 1:**
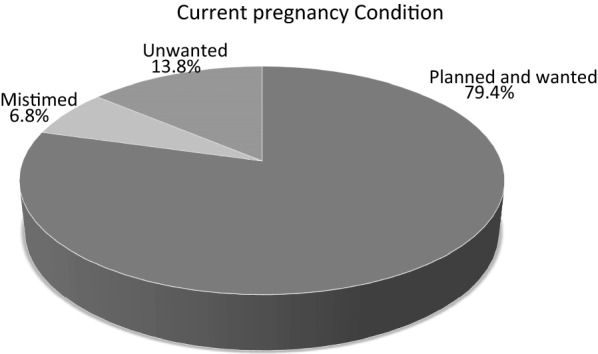
Prevalence of unintended pregnancy among pregnant women in Gondar town, North western Ethiopia, 2014

Among the total respondents 20.6% of women were their pregnancy was unintended. The rest 79.4% were intended (Fig. 1).

#### Associated factors of unintended pregnancy

Bivariate analysis was done to asses any association between independent variables and dependant variable.

In bivariate analysis: family size, women education, age, age at first pregnancy, occupation and marital status was considered statistically significant with state of pregnancy.

Multivariable logistic regression analysis showed that women age at first pregnancy < 18 years were three times more likely to commence being unintended pregnancy when compared to the counter parts (AOR [95% CI] = 3.025 (1.234, 6.056). Likewise mothers whose family size ≥ 4 were three times more likely to being the pregnancy unintended than family size < 4 (AOR [95% CI] =2.92 (1.605, 5.314) and women that have single marital were more likely the pregnancy being unintended (AOR [95% CI] =12.592 (5.182, 30.6) (Table 2).

**Table 2:** Logistic regression analysis of socio-demographic and obstetric factors for unintended pregnancy among pregnant woman in Gondar town, Northwest Ethiopia, 2014

Variables	Unintended pregnancy		COR/CI	AOR/CI
	Yes (%)	No (%)		
*Family size*				
≥ 4	38 (30.2)	88 (68.8)	2.53 (1.46, 4.38)*	2.92 (1.605, 5.314)*
< 4	29 (14.6)	170 (85.4)	1	
*Occupation*				
Waged	34 (27.6)	89 (72.4)	1.96 (1.14, 3.37)*	NS
House wife	33 (16.3)	169 (83.7)	1	
*Age, years*				
20–34	55 (19.6)	234 (81)	1	
< 20	7 (38.9)	11 (61.1)	2.71 (1.004, 7.302)*	NS
≥ 35	5 (27.8)	13 (72.2)	1.64 (0.56, 4.78)	NS
*Age at first pregnancy, years*				
< 18	75	45	5.166 (2.345, 9.042)	3.025 (1.234, 6.056)*
≥ 18	50	155	1	1
*Education*				
No formal learning	18 (24.3)	56 (75.7)	1.881 (1.002, 3.591)*	NS
Primary	25 (20)	72 (74.2)	1.741 (0.876, 3.460)	NS
Secondary and above	24 (15.6)	30 (84.4)	1	
*Marital status*				
Single	19 (67.9)	9 (32.1)	10.951 (4.675, 25.652)*	12.592 (5.182, 30.6)*
Married	48 (16.2)	249 (83.8)	1	

*NS* not associated by Backward LR model

* P-value < 0.05

### Discussion

This study showed that the prevalence of unintended pregnancy was 20.6%. The prevalence of unintended pregnancy was low in this study compared with the finding from Kersa, Hossana and Harare (29.7%, 34% and 33.3%) respectively [12–14]. This is may be due to most of the above studies were done in both urban and rural parts of the study area while our study was done only the urban part in which there is access of health center. Those women living in urban area were near by the information like different media exposure and also have clearly discuses to their parents when to have and how much child they need. The other explanation may be due to time gap; women’s awareness, knowledge about the state of pregnancy increases through time.

This finding also less than national figure of EDHS 2011 in which prevalence of unintended pregnancy among current pregnancy was 32%, this might be due to the national study addresses the large figures including both rural and urban area but our studies addresses a single urban area. Women’s who live in rural area and living in minority region may have low awareness and knowledge about the state of pregnancy because of in such area there is a limitation in information and health institutions.

In this study, socio-demographic, obstetric and information factors related to unintended pregnancy. Unintended pregnancy was significantly associated with family size. Women with family size ≥ 4 were more likely to be the pregnancy is unintended with [AOR = 2.92, 95% CI 1.605, 5.31]. This in lined with the study conducted in Jimma [15]. This might be due to an increment in family size decreases mother’s desire to have additional child there are busy to care their family and didn’t get easily the accesses of family planning and the information. Even the partner may not allow them to go the family planning service rather giving care the Childs.

The study showed that marital status had statistically significant effect on unintended pregnancy. Women with single marital status were 12.59 times more likely to report having unintended pregnancy as compared to women those were married [AOR = 12.59, 95% CI 5.18, 30.6]. This finding was supported by study in Kenya [16]. This may be due to Stigma, inadequate sexuality information and cultural pressure to appear sexually chaste and inexperienced also hinder utilization of family planning services among unmarried girls. The culture puts significant effect on this tragedy; because of those unmarried women not allowed to use contraceptive from the family because of they consider they didn’t have sex before married.

The analysis showed that respondents who were their age at first pregnancy < 18 years were more likely being unintended pregnant than whose their age at first pregnant ≥ 18 years AOR [95% 3.025 (1.234, 6.056)]; the explanation could be the fact that awareness and level of decision making increases with age and those teenagers were shamed to come the health center to get reproductive health care. And also those teenagers not allowed from their partner and family to utilize contraception due to different reasons raised by the culture.

The variables used in the study might not be exhaustive and some other variables might be missed such as: distance to health institution, partner involvement on reproductive health, religious view of family planning and cultural perspective. In addition, qualitative and large scale community based studies that can address factors of unintended pregnancy.

### Conclusion

In conclusion, even though different family planning services are available freely to all women, the prevalence of unintended pregnancy was high. Marital status, age at first pregnancy, family size was significantly associated with unintended pregnancy. As per the findings, Increase awareness of mothers on the consequence of unintended pregnancy and how to prevent it put positive mind set on modern contraceptive.

## Limitation of the study

We used small sample size which may affect the generalization to the whole targeted population.

Since the data collectors were Health professionals there may be some social desirable responses bias for some of the variables.

## Abbreviations

EDHS: Ethiopian Demography Health Survey
WHO: World Health Organization
